# Fumarate hydratase (FH) and cancer: a paradigm of oncometabolism

**DOI:** 10.1038/s41416-023-02412-w

**Published:** 2023-09-09

**Authors:** Lorea Valcarcel-Jimenez, Christian Frezza

**Affiliations:** 1 Department of Biochemistry and Molecular Biology, Faculty of Science and Technology, UPV/EHU, University of the Basque Country (UPV/EHU), Leioa, Bizkaia, Spain; 2 University of Cologne, Faculty of Mathematics and Natural Sciences, Institute of Genetics, Cluster of Excellence Cellular Stress Responses in Aging-associated Diseases (CECAD), Cologne, Germany; 3 University of Cologne, Faculty of Medicine and University Hospital Cologne, Cluster of Excellence Cellular Stress Responses in Aging-associated Diseases (CECAD), Cologne, Germany

**Keywords:** Cancer metabolism, Urological cancer

## Abstract

Fumarate hydratase (FH) is an enzyme of the Tricarboxylic Acid (TCA) cycle whose mutations lead to hereditary and sporadic forms of cancer. Although more than twenty years have passed since its discovery as the leading cause of the cancer syndrome Hereditary leiomyomatosis and Renal Cell Carcinoma (HLRCC), it is still unclear how the loss of FH causes cancer in a tissue-specific manner and with such aggressive behaviour. It has been shown that FH loss, via the accumulation of FH substrate fumarate, activates a series of oncogenic cascades whose contribution to transformation is still under investigation. In this review, we will summarise these recent findings in an integrated fashion and put forward the case that understanding the biology of FH and how its mutations promote transformation will be vital to establish novel paradigms of oncometabolism.

## Introduction

Cancer encompasses a group of diseases characterised by unrestrained cell proliferation and the ability to migrate from their original site to invade distant organs [1]. To become cancerous, cells need to acquire molecular features that pave the way to malignant transformation [2, 3], among which the reprogramming of cellular metabolism plays a key role [2]. While the first observations of metabolic alterations in tumour cells were made over a century ago [4, 5], cancer metabolism has become a topic of renewed interest since the beginning of the 21st century. Initially, most of the works supported the notion that tumour cells use nutrients differently than normal cells to fulfil the increased energy demands due to increased proliferation. We now know that the metabolic alterations occurring in tumorigenesis affect multiple layers, and not only support the nutritional status of the cells but also affect cell behaviour, including the regulation of cell signalling and mechanics [6]. Mitochondria play a crucial role in these metabolic alterations of cancer [7] and in some circumstances, their dysfunction can predispose to cancer. For instance, mutations in the enzyme Fumarate hydratase (FH) [8], Succinate dehydrogenase (SDH) [9] and Isocitrate dehydrogenase (IDH) [10], cause hereditary and sporadic tumours. In these conditions, mitochondrial metabolites accumulate, act as oncogenic signalling molecules (oncometabolites), and promote tumorigenesis [11]. Despite the progress in our understanding of the role of oncometabolites in cancer, the mechanisms underpinning cell transformation and cancer progression in these metabolically impaired tumours need further investigation. In this review, we will focus on the recent discoveries on the role of FH loss and consequent fumarate accumulation in oncogenesis. Moreover, we will discuss the future directions to improve our understanding of FH loss in cancer and potential therapeutic interventions to treat FH-deficient tumours.

### Fumarate hydratase loss and cancer

FH is a highly conserved homotetrameric cytosolic and mitochondrial enzyme expressed in most tissues, except the ovaries, vagina, muscle, adipose tissue, and bone marrow [12]. The same transcript encodes both forms, and the localization is determined by the cleavage of the N-terminal mitochondrial targeting sequence [13, 14]. In the cytosol, FH participates in pathways where fumarate is produced, such as the urea cycle and the purine nucleotide cycle (PNC). In the mitochondria, FH catalyses the reversible hydration of fumarate to malate as a step of the tricarboxylic acid cycle (TCA) cycle, also known as the Krebs cycle or citric acid cycle. FH inactivation leads to aberrant accumulation of fumarate, affecting this essential pathway and thus the production of cellular energy and generation of macro-molecular precursors [15].

Heterozygous germline mutations in FH followed by the loss of the second wild-type allele (loss of heterozygosity-LOH) predispose to hereditary leiomyomatosis and renal cell cancer (HLRCC), a syndrome characterised by benign cutaneous and uterine leiomyomata, renal cysts, and type 2 papillary renal tumours [8, 16]. Notably, renal tumours in HLRCC patients are the most aggressive form of renal cancer, characterised by early metastasis (even with a small primary tumour size) and poor clinical outcome [17]. Although pathogenic variants of FH have been identified and provide precise genetic and surveillance programs to HLRCC families, there is no association between mutation sites and clinical outcomes in patients [17–19]. Still, FH variants in HLRCC patients lead to loss of function, and the consequent LOH in the tumour tissue causes the complete loss of its enzymatic activity [20]. Therefore, cell transformation is likely caused by FH loss of enzymatic function rather than by the aberrant activity of mutant protein variants. FH loss can predispose to cancer and has been observed in other tumour types, such as paraganglioma and pheochromocytoma [21–23], adrenocortical carcinoma [24], neuroblastoma [24, 25], glioma, ependymoma, osteosarcoma and Ewing’s sarcoma [24]. Furthermore, FH is downregulated in sporadic clear cell carcinomas [26] and colorectal cancer [27], and some evidence supports the role of FH mutations in breast, bladder, and testicular cancers [16]. Still, the mechanisms underlying the oncogenic events mediated by FH loss are only partially understood.

### FH-deficient associated metabolic, molecular, and cellular reprogramming in cancer

#### Metabolic adaptations associated with FH loss

**Fig. 1:**
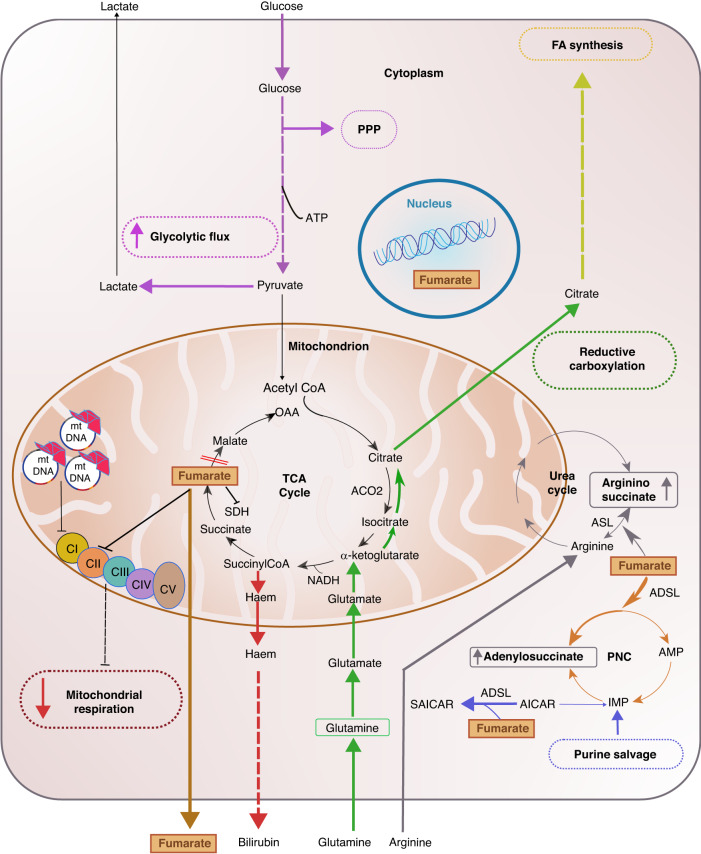
Metabolic adaptations in FH-deficient cells. FH loss leads to the truncation of the TCA cycle and subsequent accumulation of fumarate (highlighted in orange). Mitochondrial respiration is significantly reduced due to the loss of expression of mitochondrial DNA (mtDNA)-encoded subunits of the electron transport chain (ETC) and the inhibition of Succinate Dehydrogenase (Complex II of the ETC). Because of this, cells increase the glycolytic flux by taking up glucose and producing lactate to obtain energy. Furthermore, part of the carbons from glucose is diverted towards the pentose phosphate pathway (PPP) to maintain the redox homeostasis producing NADPH (purple arrows). Moreover, to maintain the remaining TCA activity FH-deficient cells increase glutamine uptake and oxidation (green arrows). On the one hand, glutamine-derived carbons are metabolized to fumarate and and bilirubin (secreted to the media) produced via the haem pathway (red arrows). On the other hand, glutamine carbons are used through reductive carboxylation to increase fatty acid (FA) synthesis (yellow arrows). Due to the accumulation of fumarate up to millimolar levels, FH-deficient cells activate multiple strategies to buffer the potential toxicity. For example, fumarate can permeate to the nucleus and also be secreted extracellularly. In the cytoplasm, fumarate accumulation leads to aberrant production of argininosuccinate via the reverse reaction of argininosuccinate lyase (ASL) in the urea cycle (grey arrows). In this context, it is important to remark that FH-deficient cells depend on a constant uptake of extracellular arginine, which becomes essential for the viability of these cells, to maintain this buffering system. Finally, fumarate can also alter PNC (Purine Nucleotide Cycle), where the increase of fumarate causes the reversal of adenylosuccinate lyase (ADSL) to form adenylosuccinate, altering de novo purine biosynthesis and making cells reliant on the salvage pathway to support purine synthesis. ATP Adenosine Triphosphate, SDH Succinate Dehydrogenase, ACO2 Aconitase2, OAA Oxaloacetate, NADH Nicotinamide adenine dinucleotide, IMP Inosine Monophosphate, AMP Adenosine Monophosphate, CI-V Electron transport chain Complex I–V), SAICAR succinyl-5-aminoimidazole-4-carboxamide-1-ribose-5′-phosphate, AICAR 5-Amino-1-(5-Phospho-D-ribosyl)imidazole-4-carboxamide.

The TCA cycle is responsible for generating intermediates for biosynthetic pathways and reducing equivalents (NADH and FADH_2_) to produce ATP through oxidative phosphorylation (OXPHOS). Besides functioning as a catabolic pathway, it also provides fatty acid and steroid biosynthesis precursors in the form of Acetyl-CoA. Moreover, it fuels gluconeogenesis and amino acid biosynthesis [28, 29]. Considering the relevance of the TCA for cell survival, it was surprising that FH loss was not only tolerated by the cells but also that it led to the development of tumours in HLRCC patients. Indeed, TCA cycle enzymes were considered indispensable for the life of a cell (see discussion in this review [30]). This observation led to the hypothesis that FH-deficient cells would need to rewire their metabolism to survive. Our group and others have investigated these metabolic adaptations in FH-deficient cells, summarised in Fig. 1. Firstly, due to the truncation of the TCA cycle and the subsequently reduced mitochondrial output induced by FH loss, cells increase their glycolytic flux and divert glucose towards lactate production for ATP synthesis and the pentose phosphate pathway (PPP) for reducing power [31, 32]. Interestingly, it has been recently described that the OXPHOS and mitochondrial dysfunction are hardwired in human lines derived from HLRCC patients in that the loss of expression of mitochondrial DNA (mtDNA)-encoded subunits of the respiratory chain complexes and mtDNA mutations cause an irreversible glycolytic switch in HLRCC patients [33]. These results differ from those observed in mouse *Fh1*-deficient cells, where the mitochondrial defects are reversed upon *Fh1* re-expression ([34]). It is possible, therefore, that mutations or loss of mtDNA observed in human lines and patient tissues accrue over several time upon the initial FH loss, and that these defects are selected to induce a more malignant glycolytic phenotype during tumour progression.

While full oxidation of glucose in the mitochondria is limited in FH-deficient cells, including anaplerosis via pyruvate carboxylase [31], glutamine becomes the main source of carbons for the truncated TCA cycle present in these cells [32]. Importantly, carbon tracing experiments using fully labelled glutamine, showed that fumarate derives from glutamine oxidation, arguing for a “forward” turning truncated TCA cycle activity in these cells [32, 35]. Whether partial TCA cycle reverse activity due to the large accumulation of fumarate, leading to SDH reversal and fumarate reduction to succinate, as it has been observed in ischaemia [36], or cells treated with mitochondrial inhibitors [37], occurs in *Fh*-1 deficient cells has not been reported. In addition, together with the production of NADPH in the PPP, glutamine supports, via the reductive carboxylation of α-ketoglutarate to citrate, fatty acid (FA) synthesis. This metabolic rewiring boosts rapid proliferation and defence against oxidative stress [31], as observed in UOK262 cells, a cell line derived from a patient with HLRCC-associated aggressive kidney cancer [38]. Of note, this process involves both IDH1 and 2 (cytosolic and mitochondrial isoforms, respectively) and is dependent on the mitochondrial aconitase (ACO2) [35, 39]. Nonetheless, reductive carboxylation in FH-deficient cells is controversial since it has been observed in human but not in mouse renal cell lines [32] or fibroblasts [40] where fumarate-mediated succination inactivates ACO2. Of note, when using an FH inhibitor, which increases fumarate but not to the same extent as observed in *Fh1*-deficient cells, mouse renal cells can still undergo reductive carboxylation [41]. These results indicate that the various FH-deficient models accumulate fumarate at different levels, affecting ACO2 function to a different degree. Consistent with this hypothesis, human FH-deficient cells accumulate lower fumarate levels than the mouse *Fh1*-deficient cells [40–42]. As reductive carboxylation plays a crucial role in conferring bioenergetics and biosynthetic advantages for tumorigenesis in cells with defective mitochondria [35], it is possible that FH-deficient cells with lower levels of fumarate are selected during tumour progression to sustain survival and tumour growth.

As glutamine supports part of the TCA cycle, NADH is generated and used by OXPHOS for ATP generation and maintenance of the mitochondrial membrane potential [32] (Fig. 1). The mitochondrial membrane potential plays a crucial role in mitochondrial homeostasis and its alterations have been associated with enhanced invasive properties and distinct functional mitochondrial heterogeneity in lung tumours [43, 44]. Interestingly, in order to maintain the carbon flux through the truncated TCA cycle, FH-deficient cells engage in the haem biosynthesis and degradation pathway, a pathway essential for their survival [32]. All these metabolic changes allow FH-deficient cells to sustain their bioenergetics, proliferative capacity, and reductive power demands enabling them to survive.

Apart from the above-described metabolic adaptations, one of the most striking biochemical and metabolic features of FH loss is the accumulation of fumarate up to millimolar levels [32, 42]. At these high levels, fumarate permeates other cellular compartments beyond the mitochondria. It can be found in the cytosol and nuclei [45–47], and be secreted extracellularly [48]. The high levels of fumarate can impact the enzymatic reactions in which it is normally involved either as a substrate or a product (Fig. 1). For instance, the urea cycle [42, 46] and the PNC [49] have been shown to be altered due to fumarate accumulation. In the urea cycle, whose rewiring has been described in cancer to support anabolism, argininosuccinate and aspartate are converted into arginine and fumarate by the enzyme argininosuccinate lyase (ASL) [50]. Fumarate accumulation can revert this reaction, producing argininosuccinate from exogenous arginine [42]. Because of this metabolic rerouting, FH-deficient cells become dependent on arginine, which is essential for the viability of the cells [42, 46]. Another metabolic pathway altered by fumarate accumulation is the PNC, where the increase of fumarate causes the reversal of adenylosuccinate lyase to form adenylosuccinate, altering de novo purine biosynthesis [19] and making cells reliant on the salvage pathway to support purine synthesis. Furthermore, it has been observed that fumarate and succinate share a similar affinity for the succinate-binding site of succinate dehydrogenase (SDH). Due to the likely high intracellular concentration of fumarate in the mitochondrial matrix, fumarate blocks the access of succinate to SDHA active site, blocking SDH-driven respiration [34].

Overall, the loss of FH leads to a coordinated metabolic rewiring that compensates for the truncation of the TCA cycle and, in parallel, allows for the buffering of excess fumarate. Importantly, the metabolic adaptations upon FH loss have been shown to be required for survival and could be used as a therapeutic strategy based on metabolic synthetic lethality [51].

#### Pro-oncogenic signalling in FH-deficient cells

**Fig. 2:**
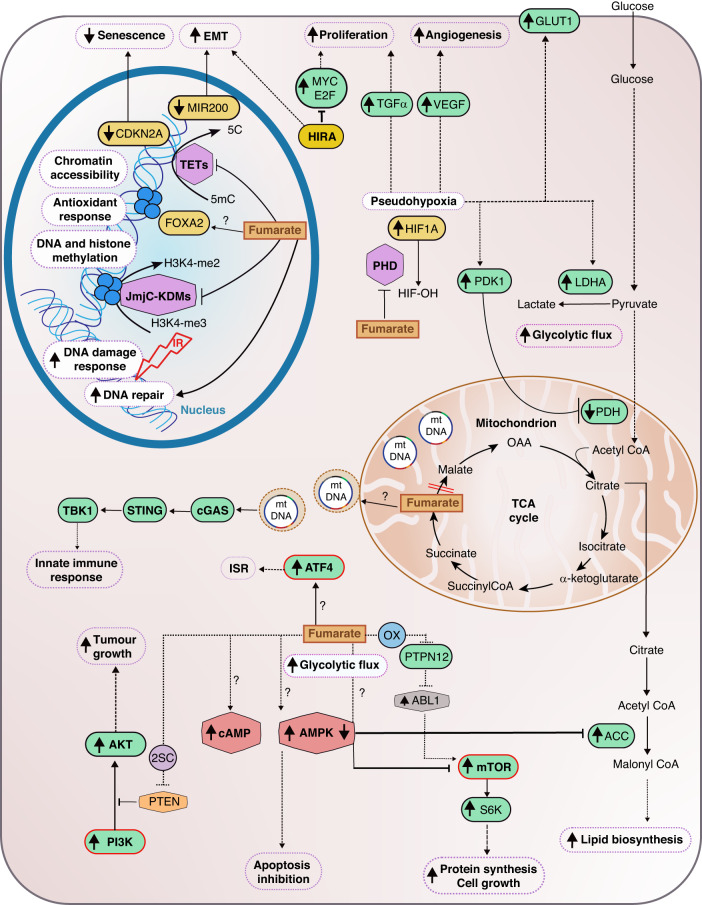
FH loss-associated oncogenic signalling. Upon FH loss, and consequently fumarate accumulation, several oncogenic pathways are altered. For example, fumarate can inhibit the activity of α-ketoglutarate-dependent dioxygenases (αKGDDs), including prolyl hydroxylases (PHDs), Jumonji C-domain lysine demethylases (JmjC-KDMs), and 10–11 translocation (TET) DNA cytosine-oxidizing enzymes. The inhibition of PHDs lead to the stabilization of hypoxia-inducible factor (HIF1A) even in normoxic conditions, known as pseudohypoxia. This phenomenon leads to the activation of signalling cascades associated with tumorigeneses, such as angiogenesis (VEGF), proliferation (TGFα) and glycolytic flux activation (activation of LDHA and GLUT1, inhibition of PDH). In the nucleus, fumarate inhibits the function of JmjC-KDMs and TETs affecting DNA and histones demethylation respectively. Specifically, the inhibition and demethylation of *miR200* and CDKN2A (p16) has been shown to trigger an epithelial-to-mesenchymal transition and to inhibit senescence respectively in HLRCC patients. In line with this, FH modulates chromatin accessibility and the activation of FOXA2-mediated antioxidant response. Beyond αKGDDs inhibition, FH loss modulates the energy sensing in the cells. For example, it has been shown to inhibit and activate AMPK function. The inhibition of AMPK can lead to the activation of lipid biosynthesis through acetyl-CoA carboxylase (ACC) and the activation of mTOR signalling. AMPK activation, instead, was shown to protect cells from apoptosis. Further evidence supports the activation of mTOR through the inactivation of ABL1, modulated by the protein-tyrosine phosphatase PTPN12. Additionally, cyclic AMP (cAMP) levels increase upon FH loss, affecting cellular energy metabolism. Beyond energy sensing, well-known oncogenic pathways have been shown to be altered in FH-deficient models. For instance, the tumour suppressor PTEN can be inhibited by fumarate through succination (2SC), activating the phosphatidylinositol-3-kinase (PI3K) cascade. Moreover, FH loss has been associated with the activation of the integrated stress response (ISR) through ATF4. Given the regulation of PI3K pathway, mTOR and ATF4 by FH loss, it is tempting to speculate a potential regulation node between them (red line). In addition, HIRA loss has been recently found to increase the tumorigenic potential of FH-deficient cells through the MYC proto-oncogene and E2F transcriptional programs. Furthermore, FH loss can also regulate and increase DNA damage response and repair upon ionising radiation (IR). Finally, it has been recently discovered that FH loss can trigger the activation of the innate immune response activating the cGAS/STING/TBK1 pathway upon mitochondrial DNA (mtDNA) release to the cytosol. CDKN2A Cyclin-Dependent Kinase Inhibitor 2A, LDHA Lactate Dehydrogenase A, GLUT1 Glucose Transporter 1, PHD Pyruvate Dehydrogenase Complex, VEGF Vascular Endothelial Factor, TGFα Transforming Growth Factor alpha, FOXA2 Forkhead Box A2, AMPK AMP-activated Protein Kinase, PTEN Phosphatase and tensin homolog, ATF4 Activating Transcription Factor 4, mTOR mammalian target of rapamycin, cGAS cyclic GMP–AMP synthase, STING Stimulator Of Interferon Response CGAMP Interactor 1, TBK1 TANK-binding kinase 1, OX Oxidation.

Many of the genetic and epigenetic alterations occurring in cancer affect signalling pathways that control and sustain cell growth and division, cell death, cell fate, and motility [2, 52]. Fumarate accumulation not only triggers metabolic alterations in the cells but also alters the function of different signalling cascades that can profoundly alter tumorigenesis (Fig. 2). Due to its electrophilic nature, fumarate can react with nucleophilic residues such as thiol groups from cysteine residues exposed at the surface of proteins to generate a stable thioether, S-(2-succino) cysteine (2SC) [53]. This post-translational modification can affect various proteins, altering signalling cascades in the cells, whose role in tumorigenesis is beginning to be understood [54, 55]. In addition to succination, whose signalling is beyond the scope of this review and has been reviewed before [56], in the next sections, we will discuss the different signalling cascades triggered by FH loss and fumarate accumulation.

##### Fumarate-mediated αKGDDs inhibition signalling

α-ketoglutarate-dependent dioxygenases (αKGDDs) are a family of more than 60 iron-containing enzymes that use α-ketoglutarate and oxygen to hydroxylate various substrates, regulating their functions. αKGDDs are activated by ascorbate (Vitamin C) and inhibited by different TCA cycle intermediates, including fumarate [57]. Fumarate has been postulated to regulate by competitive inhibition the activity of αKGDDs, including prolyl hydroxylases (PHDs), Jumonji C-domain lysine demethylases (JmjC-KDMs) and ten-eleven translocation (TET) DNA cytosine-oxidizing enzymes [57, 58] (Fig. 2).

PHDs catalyse the hydroxylation of proline residues in target proteins [57]. In normoxic conditions, PHDs hydroxylate the α-subunit of Hypoxia Inducible Factors (HIFs), facilitating their proteasomal degradation [59, 60]. In this context, it has been observed that PHDs can be inhibited by fumarate leading to the stabilisation of HIF1α and HIF2α even in normoxia, a phenomenon known as pseudohypoxia [61, 62], and the activation of HIF-associated signalling cascades such as cell growth, angiogenesis, cell survival, migration, and metastasis [63]. Indeed, several hallmarks of cancer as epithelial-to-mesenchymal transition (EMT), angiogenesis, cell adhesion, and cytoskeleton rearrangements are ascribed to HIFs activation [63, 64]. In addition, HIFs can control the expression of metabolic enzymes involved in glucose metabolism, such as lactate dehydrogenase A (LDH-A) [64], pyruvate dehydrogenase kinases (PDKs) [65], and the glucose transporter 1 (GLUT1) [63, 66]. Interestingly, these metabolic changes mainly support increased glucose uptake and glycolytic flux towards lactate, consistent with the metabolic alterations observed in FH-deficient cells (Fig. 1). Given the relevance of HIF activation in tumorigenesis, it is tempting to speculate that the fumarate-mediated HIF activation could contribute to tumour formation in HLRCC patients. Nevertheless, *Fh1*-loss-associated renal cyst formation has been shown to be HIF-independent [67], suggesting that these transcription factors, despite being crucial regulators of the response to FH loss are dispensable for transformation, at least in this model [67].

TETs are a group of α-KGDDs that catalyse the iterative oxidation of methylated cytosines on DNA, thereby facilitating DNA demethylation and controlling chromatin structure, function and transcription [57, 68]. As fumarate is able to alter the activity of these enzymes, it can affect the overall DNA methylation profile of the cells and, consequently, the expression of tumour suppressors and oncogenes [68, 69]. For example, it was shown that HLRCC patients exhibit a CpG island hypermethylation phenotype and the hypermethylation of the tumour suppressor cyclin-dependent kinase inhibitor 2A [70], encoding for p16, involved in cell cycle regulation and senescence activation [71, 72]. This result suggested that suppressing senescence could be a prerequisite for transformation in these tumours. The role of FH loss in senescence will be described in more detail in the following paragraph. Another consequence of TET inhibition mediated by fumarate is the hypermethylation and suppression of a family of antimetastatic miRNA cluster (MIR200) [73], which in turn de-repress transcription factors involved in EMT (Twist1, Zeb1, Zeb2, Snai1, and Snai2) [73], a process known to be implicated in tumour initiation and metastasis [74]. Strikingly, in nasopharyngeal carcinoma, the chromatin remodelling factor lymphoid-specific helicase triggers an EMT signature by suppressing FH [75].

Finally, another family of αKGDDs mediating epigenetic reprogramming is the JmjC-KDMs, which catalyse the demethylation of mono-, di-, and tri-methylated lysines, including on histone tails (e.g., H3K27me2/3, H3K9me2/3, H3K4me2/3) [57]. Through this mechanism, they can alter chromatin accessibility and gene expression [76]. Although the biological consequences of fumarate-dependent inhibition of histones demethylation have not been fully determined, it has been recently shown that fumarate accumulation can increase the methylation levels of H3K4, H3K27, and H3K79, inducing the activation or repression of gene transcription [77]. Consistently, a recent study has shown that FH loss modulates chromatin accessibility and allows the activation of the pioneering transcription factor FOXA2, which participates in the antioxidant response [78]. Still, further investigation is needed to understand the implications of these changes in FH-loss-associated tumorigenesis.

Interestingly, fumarate shares these “epigenome modifying” activities with succinate and 2-hydroxyglutarate, two other established aKGDDs inhibitors with oncogenic functions [76, 77]. Why these metabolites, when accumulated, give rise to different tumour types, with different tissue specificity and severity, despite having overlapping functions is still a mystery. It is possible that different inhibitory activity towards different subsets of aKGDDs, different chromatin organisation and aKGGD distribution in the cell of origin, and additional biological properties of these metabolites (such as succination for fumarate, for instance) could result in a rather specific transforming event.

##### FH loss effect on energy sensing

Beyond the above-described signalling pathways induced by fumarate accumulation, FH-deficient cells modulate additional molecular cascades that could promote tumorigenesis. Given the crucial role of FH in maintaining metabolic homeostasis in the cells, it is not surprising that energy-associated and cell fate signalling pathways are altered when FH is inactivated. One example is the modulation of the AMP-activated kinase (AMPK) [79, 80] (Fig. 2). Although AMPK is a central node maintaining energy homeostasis and orchestrating different cellular responses to nutrient stress, its role in FH-deficient cells is controversial [81]. On the one hand, in HLRCC cell lines, it was shown that the glycolytic shift occurring in FH-deficient cells and HLRCC tumours leads to the inactivation of AMPK, and the activation of anabolic factors, acetyl-CoA carboxylase and ribosomal protein S6 (effector of the mammalian target of rapamycin-mTOR), that potentially could promote oncogenic growth in renal cancer [79]. On the other hand, upon silencing of FH, AMPK was shown to be active and to protect cells from apoptosis [80]. Whether these discrepant results are due to using different cellular models, or the different effects of a chronic *vs* acute loss of FH is currently unclear.

mTOR oncogenic signalling is frequently activated in cancer, as it controls cell growth and metabolism. As observed before, the effectors of this pathway are altered in FH-deficient cells [79] (Fig. 2). Therefore, mTOR emerges as a potential player in FH-deficient tumours [82]. In line with this, the inactivation of the Abelson (ABL) murine leukaemia viral oncogene homolog (ABL1), upregulated in HLRCC tumours by inhibition of the protein-tyrosine phosphatase N12 PTPN12 [83], led to the suppression of mTOR-mediated HIF1α translation. This data highlight again the potential role of this pathway in HLRCC tumorigenesis [84]. Interestingly, the connection between FH and mTOR seems mutual: kidney-specific Tsc1 loss, which leads to constitutive mTOR activation, causes an mTOR-dependent downregulation of FH and the accumulation of fumarate, promoting renal epithelial transformation [85].

In addition to these pathways, a genome-wide RNAi screen performed in FH-deficient cells showed their dependence on adenylate cyclases. This observation was supported by increased cyclic AMP levels (cAMP) in FH-deficient cells, which may act to regulate cellular energy metabolism [86] (Fig. 2). Of note, cAMP was the first described “second messenger” whose signalling activation and modulation have been widely associated with oncogenesis and tumour progression [87]. Although the association between cAMP and FH-associated tumorigenesis may occur, this aspect has been largely unexplored and its association with cell transformation is still unclear.

Another signalling pathway commonly altered in cancer is the phosphatidylinositol-3-kinase (PI3K) cascade, whose aberrant activity is linked with human tumour progression and the invasive potential [88]. This pathway is negatively regulated by PTEN, which acts as a direct antagonist of PI3K. PTEN is a well-characterised tumour suppressor with growth, survival, and metabolic regulatory functions, and its loss has been associated with several cancers [88]. Recently, it has been shown that fumarate accumulation in HLRCC can lead to PTEN suppression through succination at cysteine C211 [89]. Hence, fumarate-dependent succination of PTEN leads to the activation of PI3K cascade, promoting growth in orthotopic renal tumours [89] (Fig. 2).

Finally, as mentioned before, FH inhibition has been associated with integrated stress response (ISR) activation through ATF4 stabilization to communicate amino acid-deprived, and redox stress state to the nucleus [41] (Fig. 2). The ISR is known to play a dual role in cancer. While it can promote a decreased proliferative rate, cancer cells can also benefit from its activation, which leads to angiogenesis, metastasis, immune cell scape, and cell stemness. The study of the activation of the ISR in FH deficiency could be relevant to understand the steps towards tumorigenesis [90].

Given the regulation at different levels of mTOR, PTEN, and ATF4 in FH-deficient tumours, it is tempting to speculate a potential link between these factors. PTEN, as a regulator of the PI3K pathway, controls AKT activation and, consequently, mTOR activation. Moreover, mTOR has been shown to alter proliferative capacities and induce metabolic adaptation through ATF4 expression and post-transcriptional control of proteins [91, 92]. Therefore, although all these pathways have been involved in FH-deficiency and tumorigenesis, it would be crucial to ascertain to what level they are independent mechanisms or cooperative events. Moreover, if this control is mutual, it would be interesting to find the triggering event activating these pathways.

##### Genomic instability, DNA repair, and senescence in FH-deficient cells

FH has also emerged as an important player in DNA damage and instability. Indeed, cells lacking cytosolic FH are more sensitive to inducers of double-strand breaks [93] (Fig. 2). Furthermore, high fumarate levels decrease homologous recombination repair (HRR) efficiency, increasing endogenous DNA damage [94]. In contrast, FH nuclear activity has been linked to the non-homologous end joining DNA repair, through FH phosphorylation upon double-strand breaks, allowing FH to bind the histone variant H2A.Z [95]. Finally, FH loss has also confer resistance to DNA damage caused by ionising radiation (IR), promoting early mitotic entry by suppressing G2 checkpoint maintenance even in the presence of unrepair damage [96].

As previously mentioned, fumarate accumulation has been associated with senescence and, therefore, cell cycle arrest^66^. For instance, it has been shown that fumarate accumulation induces persistent oxidative stress and cellular senescence in vitro and in vivo through p21 activation, a key senescence factor [97]. Remarkably, the ablation of p21 in *Fh1*-deficient mice resulted in the transformation of benign cysts into hyperplastic lesions [97]. These data, together with the epigenetic suppression of p16 (see above and Fig. 2), suggest that fumarate-induced senescence must be bypassed for full-blown transformation. However, recent data has shown that senescence is not a feature of FH loss in immortalised epithelial kidney cells and, despite not being senescent, these cells cannot form tumours in vivo [97, 98]. Interestingly, the heterozygous ablation of FH in rat fibroblasts increased p53 and TERT (telomerase reverse transcriptase), and decreased p21 and p16 expression associated with an anti-senescence phenotype [99]. In conclusion, senescence bypass is not sufficient to promote transformation and additional factors are required. To address this conundrum, a whole-genome CRISPR/Cas9 screen recently performed in *Fh1*-deficient cells has identified the histone cell cycle regulator (HIRA) as a target that, when ablated, increases proliferation and invasion in vitro and in vivo [98]. Interestingly, HIRA loss in *Fh1-*deficient cells leads to tumour initiation and growth in the kidney capsule and activates oncogenic transcriptional programs such as EMT, E2F and the proto-oncogene MYC signatures [98]. The activation of MYC in *Fh1-*deficient cells, which was previously hypothesized [100], led to increased expression of the karyopherin subunit alpha 2 (*Kpna2*), a nuclear transporter involved in the nucleocytoplasmic transport of several tumour-associated factors [98, 101] (Fig. 2).

##### FH loss controls inflammatory responses

Inflammation-associated signalling cascades, through cytokine production by innate and adaptive immune cells, can promote or suppress tumour progression, affecting therapeutic outcomes [102]. Interestingly, recent studies suggest that tumour-promoting inflammation can also be triggered by tumour cell-autonomous mechanisms^97^ that enhance the proliferative potential and tumour initiation [103]. Strikingly, a recent study by our group has shown that the inducible loss of *Fh1* led to early alterations of mitochondrial morphology and the release of mtDNA to the cytosol through mitochondrial-derived vesicles and subsequent activation of the innate immune response. This process, mediated by fumarate, activates the cyclic GMP–AMP synthase (cGAS)—stimulator of interferon genes (STING)–TANK-binding kinase 1 (TBK1) pathway [104]. Importantly, these pathways were also observed to be activated in HLRCC patients [104] (Fig. 2). In line with this, FH-deficient RCC, especially metastatic lesions, have been shown to be highly immunogenic, characterised by increased tumour T-cell infiltration but high expression of immune checkpoint cytokines [105, 106]. Interestingly, a recent genomic study has shown that metastatic lesions in these tumours display hypomethylated chemokine and immune checkpoints-related genomic loci [106]. In contrast, recent data suggest that fumarate accumulation in tumour interstitial fluid leads to inhibition of functional CD8 + T-cell activation, functioning as a metabolic barrier for the anti-tumour function of these cells [107]. Determining the effect of inflammation mechanisms and their control in FH-deficient tumours will be essential for targeted treatments.

### Relevance of co-occurring oncogenic events in FH-deficient models

We have previously discussed the metabolic and signalling cascades altered upon FH loss. However, a question remains: how are these cascades activated over time during tumour initiation and progression? It has been previously postulated that tumorigenesis driven by FH loss occurs via a two-step mechanism. First, cells need to adapt metabolically to FH loss and survive the high intracellular concentration of fumarate. Second, a series of oncogenic signalling and metabolic rewiring elicited by fumarate gradually leads to transformation [56]. This hypothesis has been recently supported by a study, in which it was shown that during the initial phase, the loss of FH leads to decreased proliferation and DNA damage. In the subsequent phase, cells regain the ability to proliferate by acquiring adaptive mutations in pro-survival oncogenic signalling cascades, such as MAPK, Wnt, and JAK/STAT [108]. Although these adaptive mutations have not been confirmed in HLRCC patient genomic data and would need to be validated in vivo to fully confirm the impact on them in disease initiation and progression, these results are supportive of a multi-step tumorigenesis process in FH-deficient cells.

Despite the potential relevance of all the pathways previously described and associated with tumorigenesis, it is important to remark that kidney-specific *Fh1*-loss does not generate tumours in vivo, but only pre-malignant cysts [109]. Therefore, FH loss may contribute partially or permits tumorigenesis rather than initiates it. A good example of this is the activation of a chronic inflammatory response in an inducible model of *Fh1*-loss through the cGAS/STING pathway [104]. While acute inflammatory reactions stimulate anti-tumour immune responses, chronic inflammation facilitates tumour progression and treatment resistance [102]. In line with this, FH inhibition in LPS-stimulated macrophages leads to the activation of non-cell autonomous innate immunity through interferon activation [110]. Therefore, the innate immune response mediated by fumarate accumulation could enhance the permissiveness of the cells to acquire oncogenic events. Furthermore, according to the recent discovery of mtDNA mutations in HLRCC patients [33], it is important to consider potential mutations and alterations in mitochondrial DNA content affecting tumorigenesis in these tumours. Indeed, mtDNA has been shown to be a major source of driver mutations in cancer [111].

Additional novel oncogenic events controlled by FH loss have been identified. Indeed, FH has been shown to be a regulator of hematopoietic stem cell functions [112]. In this case, fumarate acts as an inhibitor of leukemic transformation in acute myeloid leukemia (AML), although it is not required for disease maintenance. Specifically, self-renewing hematopoietic stem cells require *Fh1* and the capacity for maximal mitochondrial respiration to maintain their pool and Meis1/Hoxa9-driven leukemic propagation [112]. Interestingly, fumarate accumulation-mediated succination of LYN (tyrosine protein kinase) and consequent inhibition of the B-cell antigen receptor signalling, could affect the progression of B cell leukaemia and lymphomas [113, 114]. In line with this, a recent study aiming at identifying genes, mutations and biological processes that give selective advantage to mutant clones in blood cancer, identified FH to harbour copy-neutral loss of heterozygosity. These mutations across the genome promote the expansion of haematopoietic cells, increasing the polygenic drive for blood-cell proliferation traits [115].

Finally, tumours in the kidney capsule have been only observed when injecting FH-deficient cells but not in autochthonous models of FH loss. Of note, these tumours originated using UOK262 cells and/or altering additional oncogenic events, such as the inhibition of the tumour suppressor PTEN through succination, or the depletion of HIRA. In this context, it would be interesting to analyse whether somatic mutations occur in these tumour suppressors and proto-oncogenes in HLRCC patients. Still, as HLRCC tumours are rare and there is a lack of genetic information, it is unknown whether these oncogenic events occur due to additional genetic mutations, or if they are a direct consequence of FH loss. Besides the possibility that a yet-to-be-identified oncogenic event could explain the failure to generate a mouse tumour model of HLRCC, other explanations for this discrepancy could be considered. For instance, the lack of appropriate mouse models of HLRCC can be attributed to the intrinsic differences between mouse and human aetiology of renal cancer. In addition, it is possible that the current mouse model, which capitalises on the Ksp-CRE, may be targeting an inappropriate tubular subpopulation. Indeed, it is possible that by using different lineage-specific promoters for CRE-recombinases, tumours could be observed. Therefore, improved in vivo models of HLRCC and papillary type 2 renal cancer are needed to ascertain the factors promoting tumour initiation, progression, and permissiveness in FH-deficient tumours.

### Future perspectives towards therapeutic intervention of HLRCC

**Fig. 3:**
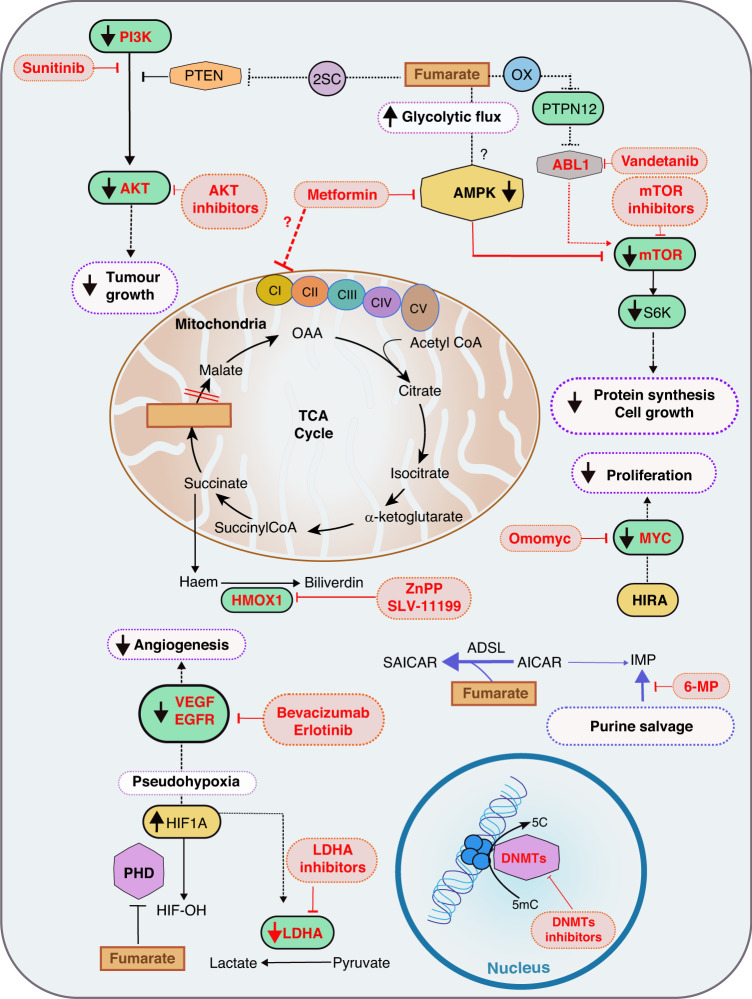
Therapeutic intervention for HLRCC patients. Given the control that FH loss and fumarate accumulation exert on several oncogenic factors, multiple inhibitors are being or could expect to be used in clinical interventions. For instance, PI3K cascade activation can be targeted using Sunitinib and the downstream effects of it using AKT inhibitors. Moreover, AMPK is re-activated through metformin treatment, indirectly inhibiting mTOR activation. Still, metformin could affect the complex I in the electron transport chain. As FH deficiency leads to decreased oxidative phosphorylation (OXPHOS), it is unclear whether this treatment could be beneficial. Beyond metformin, mTOR activation could be targeted by ABL1 inhibitors. Metabolically, these tumours could benefit from HMOX1 inhibition using zinc protophorphyrin (ZnPP) or an imidazole-based inhibitor SLV-11199, and LDHA inhibitors affecting the glycolytic flux. In addition, the inhibition of the purine salvage pathway using 6-mercaptopurine (6-MP) has been shown to affect the viability of FH-deficient cells. Furthermore, several anti-angiogenic therapies based on VEGF and EGFR inhibition are currently being used in the clinic. Interestingly, the new results highlighting the role of MYC activation in these tumours could open new therapeutic strategies based on a novel MYC inhibitor, omomyc. Finally, given the alterations occurring in the epigenetic machinery in FH-deficient tumours, inhibitors of DNA methyltransferases (DNMTs) are being tested in clinical trials. PI3K Phosphatidylinositol-3-kinase, AKT AKT Serine/Threonine Kinase 1, ABL1 ABL Proto-Oncogene 1, mTOR mammalian target of rapamycin, AMPK AMP-activated Protein Kinase, PTEN Phosphatase and tensin homolog, HMOX1 Heme Oxygenase 1, VEGF Vascular Endothelial Factor, LDHA Lactate Dehydrogenase A, EGFR Epidermal Growth Factor Receptor, 2SC 2-succinic-cysteine, CI-V Electron transport chain Complex I–V, HIF1A Hypoxia Inducible Factor A, PHD Prolyl hydroxylase.

As briefly described in this review, FH loss leads to a wide range of oncogenic events. The dysregulation of energy homeostasis leads to the activation of key oncogenic pathways and transcriptional programs, such as the ones controlled by HIF, mTOR, and PI3K. Furthermore, the multifaceted change in mitochondrial processes elicits inflammatory responses and the ISR. Interestingly, FH-associated tumorigenesis is enhanced by the activation of well-known proto-oncogenes, such as MYC, and suppression of tumour suppressors, including PTEN. Despite substantial advances in understanding HLRCC aetiology, several aspects of FH biology remain to be addressed. As mentioned in the previous sections, the role of FH loss and fumarate on the immune system, as well as other components of the tumour microenvironment, need further investigation. For instance, how FH-deficient cells influence the tumour microenvironment and communicate with surrounding cells, possibly shaping immunity and inflammation will be a very important future line of investigation. It would also be important to understand why FH loss gives rise to such a unique set of tumours, affecting different tissues with different malignancy. Revealing the metabolic determinant of this tissue specificity could help to devise new strategies for tumour prevention, based on selective nutrient deprivation aimed at targeting tissue-specific pro-survival metabolic adaptations. Finally, and likely the most clinically relevant challenge will be targeting some of the hallmarks of FH loss for cancer therapy. Some recent findings show that this is becoming possible (Fig. 3). For instance, the stabilization of HIF in HLRCC patients results in the upregulation of several pro-survival pathways, including angiogenesis [61–63]. Preliminary data from phase II clinical trials suggest that targeting downstream targets of HIF can be useful in patients with HLRCC [116, 117]. Consistently, combination therapy with bevacizumab, a VEGF inhibitor, and erlotinib, an EGFR inhibitor has been proposed [117]. In addition, we have seen that AMPK levels decrease upon the metabolic shift generated by FH loss [79]. The attenuation of AMPK leads to the activation of mTOR and cell proliferation. As observed in other diseases such as type 2 diabetes, metformin can be used to re-activate AMPK, and, therefore, could be a promising approach to treat FH-deficient tumours. Still, as metformin inhibits the respiratory chain complex I, and FH deficiency leads to a decreased OXPHOS activity, it is unclear whether this treatment would be beneficial [118]. mTOR activation has also been targeted in these patients, although its sole action seems not to have any effect and needs to be combined with VEGF inhibitors [116]. In line with this, vandetanib, a tyrosine kinase inhibitor targeting ABL1, functions as a synthetic lethal factor in FH-deficient cells suppressing mTOR-mediated HIF1α translation [83]. Moreover, additional tyrosine kinase inhibitors, such as sunitinib, and AKT inhibitors could be a useful HLRCC therapy in the context of PTEN inhibition [89]. Another synthetic lethal factor in FH-deficient cells is Hmox1, whose inhibition with zinc protophorphyrin (ZnPP) or an imidizaole-based inhibitor SLV-11199, leads to decreased cell growth [32, 119]. Finally, the proto-oncogene MYC, whose activation boosts tumorigenesis could be used as a potential therapeutic target. Indeed, MYC inhibition has been shown to be an effective anti-oncogenic therapeutic strategy. Omomyc, a dominant mutant interfering Myc bHLHZip dimerization domain, has been shown to revert cell transformation in vitro and Myc-driven tumorigenesis in vivo [120].

Given the defects in TCA cycle activity upon FH loss [41], future treatments could also target the broad metabolic changes. For instance, LDH-A inhibition was shown to increase apoptosis in vitro and diminish xenograft tumour growth in vivo [64]. In line with this, as FH-deficient cells show a high dependency on arginine, a potential treatment approach could also be arginine deprivation [42]. Furthermore, given the dependence of these cells to the purine salvage pathway, using 6-mercaptopurine, the cell viability could be affected [19]. Interestingly, the robust metabolic rewiring occurring in FH-deficient cells has been recently exploited to identify non-invasive plasma biomarkers suitable for rapid diagnoses. In particular, two tumour-derived metabolites, succinyl-adenosine and succinic-cysteine were found to faithfully reflect FH mutation status and tumour mass [121]. These findings are crucial for rapid diagnosis and screening, to improve clinical outcomes and therapeutic strategies for patients.

Moreover, as FH-deficient tumour models and HLRCC samples show an inflammatory response, these tumours may benefit from PD1/PDL1 inhibitors. Indeed, it has been observed that HLRCC tumours, associated with high intratumoral PD-L1 and lymphocytes PD-1 expression, had the most pronounced inflammatory infiltrate of both CD4 and CD8T cells [122]. In addition, there are some case reports of immune checkpoint inhibitors showing promising results for HLRCC treatment. Still, there is minimal information on the potential therapeutic implications for HLRCC patients. Finally, there are some clinical trials in phase II to assess the safety and efficacy of PD-1 inhibitors as second-line treatment for HLRCC alone and in combination with VEGFR1/2/3 inhibitors. Therefore, although no current treatments are set, the effect of these inhibitors may be promising for HLRCC treatment [123].

Ultimately, gene-therapy-based strategies for the replacement of defective enzymes in target tissues or targeting epigenetic machinery could decrease the oncogenic impact of FH loss in HLRCC patients [116]. Indeed, inhibitors of DNA methyltransferases, a family of enzymes that add methyl groups to cytosine residues of DNA, are currently being tested in clinical trials. This treatment could have a profound impact given the DNA hypermethylation observed in FH loss [124].

In conclusion, the recent discoveries underpinning the role of FH loss in cancer open new therapeutic strategies to treat more efficiently these tumours and enable us to have a better understanding of the biology behind this rare cancer.
